# cROSsing the Line: Between Beneficial and Harmful Effects of Reactive Oxygen Species in B-Cell Malignancies

**DOI:** 10.3389/fimmu.2020.01538

**Published:** 2020-07-21

**Authors:** Krzysztof Domka, Agnieszka Goral, Malgorzata Firczuk

**Affiliations:** Department of Immunology, Medical University of Warsaw, Warsaw, Poland

**Keywords:** oxidative stress, ROS, B-cell malignancies, immune evasion, CLL, B-ALL, lymphoma, leukemia

## Abstract

B-cell malignancies are a heterogeneous group of hematological neoplasms derived from cells at different stages of B-cell development. Recent studies revealed that dysregulated redox metabolism is one of the factors contributing to the pathogenesis and progression of B-cell malignancies. Elevated levels of oxidative stress markers usually correlate with the advanced stage of various B-cell malignancies. In the complex tumor microenvironment, reactive oxygen species affect not only malignant cells but also bystander cells, including immune cells. Importantly, malignant cells, due to genetic dysregulation, are able to adapt to the increased demands for energy and reducing equivalents via metabolic reprogramming and upregulation of antioxidants. The immune cells, however, are more sensitive to oxidative imbalance. This may cause their dysfunction, leading to immune evasion and tumor progression. On the other hand, the already imbalanced redox homeostasis renders malignant B-cells particularly sensitive to further elevation of reactive oxygen species. Indeed, targeting antioxidant systems has already presented anti-leukemic efficacy in preclinical models. Moreover, the prooxidant treatment that triggers immunogenic cell death has been utilized to generate autologous anti-leukemic vaccines. In this article, we review novel research on the dual role of the reactive oxygen species in B-cell malignancies. We highlight the mechanisms of maintaining redox homeostasis by malignant B-cells along with the antioxidant shield provided by the microenvironment. We summarize current findings regarding therapeutic targeting of redox metabolism in B-cell malignancies. We also discuss how the oxidative stress affects antitumor immune response and how excessive reactive oxygens species influence anticancer prooxidant treatments and immunotherapies.

## Introduction

Oxidative metabolism is vital for all aerobic organisms. Reactive oxygen species (ROS) formed during the oxidative metabolism are important signaling molecules. However, to maintain redox homeostasis, the levels of ROS must be tightly controlled. Oxidative stress is defined as an imbalance between the generation or uptake of ROS and their scavenging. Exaggerated, prolonged oxidative stress leads to the oxidative macromolecule damage and cell death.

Dysregulated redox homeostasis is a hallmark of cancer and may lead to higher steady-state levels of ROS in cancer cells (1, 2). This is caused by the elevated production of ROS due to oncogene activation and intensified oxidative metabolism in cancer cells, as well as by ROS generated extrinsically, within the tumor microenvironment (TME). Although dysregulation of redox homeostasis has been mainly studied in solid tumors, it also occurs in hematological malignancies. Increased levels of ROS were demonstrated in many B-cell malignancies including B-cell acute lymphoblastic leukemia (B-ALL), chronic lymphocytic leukemia (CLL), and B-cell lymphomas (3–6). Noteworthy, B-cell-derived malignant cells are particularly sensitive to prooxidant treatments, which may be caused by their specific metabolic reprogramming (7, 8) or reliance on selected antioxidant pathways (9, 10). Recent studies revealed that also the TME shapes the redox homeostasis. Selected stromal cells of the TME, e.g., mesenchymal stem cells, aid malignant cells to alleviate oxidative stress, supporting their survival (11, 12). Other cells, such as macrophages or polymorphonuclear subset of myeloid-derived suppressor cells (PMN-MDSCs), generate high amounts of ROS, contributing to elevation of ROS levels in the TME (13, 14). This may negatively affect the function of effector immune cells, causing impairment of antitumor immune response.

**Figure 1:**
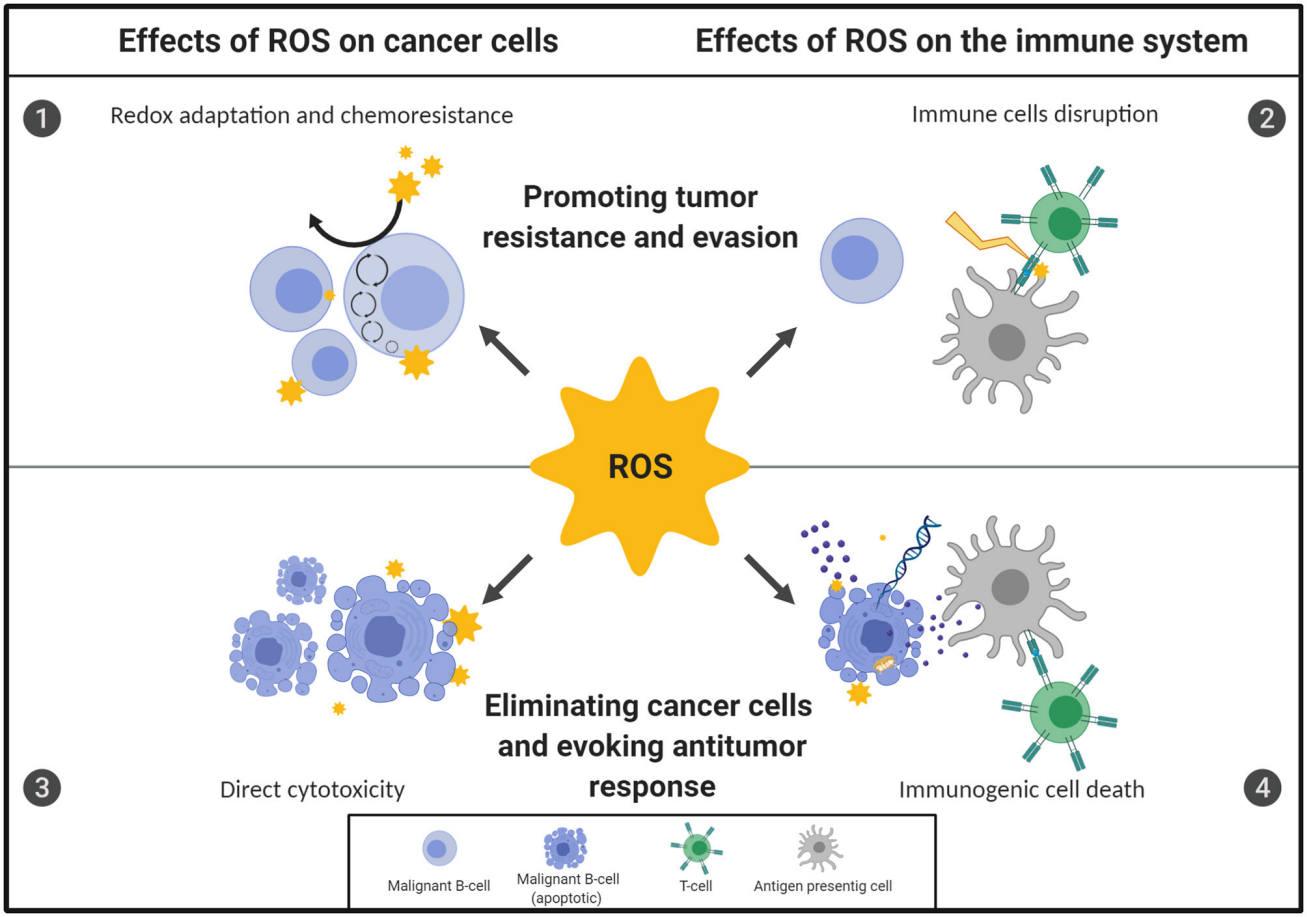
Oxidative stress displays a dual role in the fate of B-cell malignancies. The ultimate outcome of redox imbalance on B-cell malignancies depends on the source, concentration, location of reactive oxygen species (ROS), as well as the duration of exposure. Importantly, the overall effect of ROS on the fate of B-cell neoplasms depends on the action of ROS both on malignant B-cells and on the cells of the TME, including immune cells. However, malignant cells adapt to the increased demands for energy and reducing equivalents by increasing their antioxidant capacity and rewiring their metabolic energy pathways, which renders them therapy-resistant [1]. At the same time, the highly oxidative microenvironment may negatively affect function of the effector immune cells, causing impairment of antitumor immune response, contributing to immune evasion and further tumor progression [2]. Conversely, targeting specific antioxidant pathways in malignant B cells or inhibiting the antioxidant support provided by the TME trigger direct, oxidative-stress mediated cytotoxicity and can effectively eradicate malignant B cells [3]. Moreover, some forms of oxidative stress-mediated cell death under certain conditions are immunogenic. This leads to the activation of immune cells and subsequent development of antitumor response, what further contributes to the efficacy of prooxidant treatments [4]. The figure was created using BioRender.com.

Here we review novel findings of the manifold effects of steady-state and therapy-induced ROS on B-cell malignancies, including their direct influence on malignant and normal cells' survival and/or function. We also discuss the possible consequences of increased ROS levels on the effectiveness of anticancer therapies, mainly prooxidant treatments and immunotherapies. The multifaceted effects of ROS on B-cell malignancies are presented in Figure 1.

## Intrinsic and Extrinsic Sources of ROS in B-Cells Malignancies

Elevated ROS levels and subsequent oxidative stress are features of various B-cell malignancies, which in majority of cases correlate with the clinical stage of the disease and patients' survival. The accumulation of oxidation products of phospholipids and/or DNA in serum as well as elevated levels of intracellular ROS in malignant B cells were observed in patients suffering from CLL (4, 15, 16). Recently, the increased levels of oxidative stress biomarkers were also found in B-ALL (6) and different types of non-Hodgkin lymphoma (NHL) (3).

ROS come from a number of sources and are generated both by malignant cells (intrinsic) as well as the cells of the TME, nutrients, pollutants or therapies (extrinsic). Intrinsic ROS are by-products of metabolic processes such as mitochondrial oxidative phosphorylation, fatty acid oxidation (FAO), and protein folding in the endoplasmic reticulum. In humans there are over 40 different ROS-generating enzymes including various NADPH oxidases (NOXs), cytochrome P450, superoxide dismutases (SOD), and others (17–19). In CLL, the elevated ROS have been linked to increased mitochondrial biogenesis and activity (4). Although NOXs are not responsible for ROS overproduction in CLL cells (4), NOX5 is specifically expressed in hairy cell leukemia and generates H_2_O_2_, which promotes oncogenic signaling (20). High amounts of ROS also come from FAO occurring in peroxisomes and mitochondria. The lipoprotein lipase along with peroxisome proliferator activated receptor (PPAR)α are elevated in CLL, drive FAO and reprogram malignant cells to use lipids as an alternative energy source (21, 22). Other types of B-cell malignancies also display enhanced FAO. For instance, a subtype of diffuse large B-cell lymphoma (DLBCL) depends on oxidative phosphorylation and FAO for its survival and growth (23).

In addition to enhanced ROS generation, the oxidative stress in B-cell malignancies results from diminished capacity of antioxidant systems (24). Indeed, decreased activity of SOD2 and catalase (CAT) has been reported in CLL and DLBCL patients compared to healthy individuals (16, 25). Reduced CAT expression along with increased amounts of mitochondria were found in less aggressive CLL subtype (26).

Apart from cancer cells, other cells of the TME, in particular myeloid cells, may contribute to ROS production, mainly via NOX-dependent manner (27, 28). The accumulation of selected populations of monocytes and their immunosuppressive reprogramming were reported in CLL patients (29, 30) as well as in the murine Eμ-TCL1 model of human CLL (31). Moreover, Manukyan and co-workers observed functional alterations in circulating neutrophils in CLL patients including phenotype change and elevated ROS production (32).

## How Malignant B-Cells Cope With Elevated ROS Levels?

The effects of ROS on cancer cells vary depending on their concentrations (33). Low ROS levels may activate malignant B-cell proliferation through stimulation of B-Cell Receptor (BCR) and pre-BCR signaling (34), while higher levels induce apoptosis. To prevent oxidative stress and subsequent cell death due to elevated ROS levels, cells utilize numerous antioxidants. They include both small non-enzymatic molecules, such as glutathione (GSH), bilirubin, ferritin, NADPH, as well as antioxidant enzymes, e.g., thioredoxins (TXNs), thioredoxin reductases (TXNRDs), peroxiredoxins (PRDXs), CAT, SODs, heme oxygenase (HO-1) and a plethora of GSH-dependent enzymes (35, 36). The renewal of reduced GSH, TXN and other antioxidant molecules relies on cellular supplies of NADPH, which is primarily generated via pentose phosphate pathway (PPP).

Various transcription factors controlling redox balance are operating in selected B-cell malignancies. Signaling related to nuclear factor erythroid 2-related factor 2 (NRF2), a master regulator orchestrating antioxidant response, is enhanced in primary CLL (37) and mantle cell lymphoma (38) cells. Enzymes most frequently activated in B-cell malignancies belong to the TXN family. PRDX1 and PRDX2 are upregulated in Burkitt lymphoma cells (39), PRDX1 and PRDX3 in primary CLL cells (10, 40), PRDX1 and TXN1 in B-ALL cells (6), and TXN1 in the OXPHOS-DLBCL (41). The increase of antioxidant response in B-ALL occurs also via serine/threonine-protein phosphatase 2A (PP2A), which negatively regulates protein kinase B (AKT) and thus activates other key transcription factors promoting antioxidant response—the Forkhead box proteins (FOXO1 and FOXO3a). The antioxidant capacity in the B-ALL cells is further supported by NADPH production via activation of PPP, which is also mediated by PP2A (8).

## TME and Stromal Support in Maintaining Redox Homeostasis

Bone marrow niche plays a crucial role in survival and progression of B-cell malignancies. Primary CLL and B-ALL cells undergo apoptosis when grown *in vitro* without stromal support (40, 42). The co-cultures with stromal cell lines, primary mesenchymal stem cells (MSC) (6) or adipocytes (43), promote survival of primary CLL and B-ALL cells and increase their resistance to therapies (43, 44). Tumor-stroma interactions occur on many levels (45). Recent studies highlight the key role of stromal cells in alleviating oxidative stress in malignant B-cells (40). The stromal support can be delivered directly, by providing antioxidants, or indirectly, by inducing antioxidant response in malignant B-cells.

It has been found that TXN1 secreted by stromal cells in the CLL lymph nodes, promoted proliferation and survival of the primary CLL cells (12). In another study, the MSC in the bone marrow aided CLL cells by uptake of cystine via Xc- transporter and subsequent secretion of cysteine, which was then used by malignant cells to synthetize GSH and overcome oxidative stress conditions (11). The depletion of the external cysteine by recombinant cysteinase in the Eμ-TCL1 mice resulted in significantly prolonged median survival time of the mice, confirming the crucial role of the MSC-derived cysteine in leukemia progression (46). Similarly, a dependence on stromal cysteine support was also reported in B-ALL (47). The mechanisms of stromal redox support in lymphomas are less thoroughly documented, although there is some evidence that the DLBCL cells may be aided by GSH received from fibroblastic reticular cells (48).

Stromal cells can also reduce oxidative stress and protect from ROS-inducing chemotherapy by transfer of organelles to leukemic cells via tunneling nanotubes (TNTs). These cellular extensions act as bridges between cancer and stromal cells that enable intercellular transport (49, 50). Activated stromal cells transmitted mitochondria to B-ALL cells using TNT and protected B-ALL cells from cytarabine-induced apoptosis (44). However, the exact mechanism of this protection remains unclear. Presumably, it is associated with triggering of adaptive antioxidant signaling.

By comparing the transcriptomes of primary CLL cells grown in a monoculture or a co-culture with HS5 stromal cells, Yosifov et al. observed a significant differences in the expression of genes involved in ROS generation, ROS detoxification, and hypoxic signaling (40). Noteworthy, the CLL samples displaying the “co-culture-like” gene expression signature correlated with significantly worse patients' survival (40).

Alleviation of oxidative stress in the leukemic niche can also occur as a result of communication between malignant cells and stromal cells using extracellular vesicles. B-ALL cells metabolically reprogrammed stromal cells via secretion of extracellular vesicles, switching their main energy pathway from oxidative phosphorylation to aerobic glycolysis (51). Such alterations are likely to favor tumor survival by reducing oxidative stress in the microenvironment. A similar mechanism of exosome-driven metabolic reprogramming has also been discovered in CLL (52).

## Therapeutic Targeting of Redox Pathways in B-Cell Malignancies

The dependence of malignant B-cells on antioxidants can be utilized in therapy. Treatments based on the generation of excessive ROS, so called “prooxidant,” are selectively toxic to malignant B-cells and some of them exert antitumor effects *in vitro* and *in vivo*, mainly in preclinical models. Inhibition of the TXN system with auranofin or SK053 selectively killed malignant B-cells and displayed anti-leukemic activity in animal models of CLL (53) and B-ALL (6). Some efficacy against B-cell malignancies has been also demonstrated for agents interfering GSH and L-Cys (46, 54). Although blocking of a single antioxidant pathway may result in some toxicity to malignant B-cells, it is usually at best partially effective (6, 53). Inhibitors of the antioxidant systems more potently kill malignant B-cells when administered in combinations with other drugs, and particularly so in combination with ROS-generating agents such as L-ascorbate (10).

Another way to evoke excessive oxidative stress in malignant B-cells is to block the antioxidant shield provided by the microenvironment. Approaches which proved effective in preclinical models include inhibition of Xc- cystine transporter with sulfasalazine (55) as well as blocking of TNTs formation and transfer of mitochondria to B-ALL cells by inhibiting microtubule polymerization with vincristine (44). The recently presented, unbiased approach aimed to find agents capable of blocking stroma-mediated protection identified ouabain and emetine, drugs perturbing redox homeostasis. Along with the increase in mitochondrial ROS levels in CLL cells and depletion of cellular NADPH pool, emetine delayed CLL development in Eμ-TCL1 murine model (40).

The important feature of prooxidant approaches is their substantial B-lineage selectivity. Although the underlying mechanisms are not fully understood, some explanations were proposed. It was shown that thiol-reactive compounds activated NRF2-mediated signaling less extensively in malignant and normal B-cells as compared to other subpopulations of leukocytes and were more cytotoxic to CLL cells (37). The unique sensitivity of B-cells to dysregulation of redox homeostasis has been recently attributed to B-cell-specific metabolic reprograming. B-lymphoid transcription factors PAX5 and IKZF1 were shown to repress the key enzymes of the PPP, which resulted in insufficient generation of NADPH. To salvage oxidative stress, malignant B-cells upregulate PP2A, a key enzyme which redirects glucose metabolism to PPP. Accordingly, inhibition of PP2A delayed development of several different B-cell malignancies and had little effect on myeloid leukemia (8).

## How Oxidative Stress Affects Immune Response?

The effects of ROS on immune system are at least two-sided. Prooxidant therapies are potent inducers of immunogenic cell death (ICD) in malignant cells. However, prolonged oxidative stress may also negatively affect the functions of selected populations of immune effector cells. The concept of ICD has altered the traditional view of non-immunogenic apoptosis and revealed that some forms of cell death better stimulate immune response than others. In the cancer field, dying cells undergoing ICD elicit robust, coordinated innate and adaptive immune response against tumor-specific antigens. ICD and the molecular processes that define immunogenicity are summarized here (56). A number of prooxidant treatments such as ionizing radiation, photodynamic therapy, ROS and endoplasmic reticulum stress-inducing chemotherapy or drugs interfering with cancer-specific energy metabolism, have been tested for their ability to induce ICD and stimulate antitumor immune response against different types of cancers [for further reading see (57–59)]. Only some of these ROS-inducing treatment modalities have been investigated in B-cell malignancies. In CLL cells, the PPARα antagonists increased TNFα and decreased IL-10 release, as well as stimulated T cell proliferation in allogenic mixed lymphocytic reaction (22). However, the most extensively tested in the context of triggering immunogenicity of B-cell malignancies is ionizing radiation. As demonstrated in murine models of B-cell lymphoma, the time of local irradiation delivery has significant impact on the immunogenicity and the overall treatment efficacy. Only the irradiation delivered in an accelerated mode triggered ICD, increased infiltration of CD4^+^ and CD8^+^T cells, dendritic cells (DC), decreased the number of regulatory T cells (Tregs) in the TME and resulted in tumor regression (60). Ionizing radiation has been also used to generate antitumor vaccines. In NHL cell lines, ionizing radiation combined with heat shock, promoted expression of genes associated with antigen processing and presentation. The treatment was used to generate DC-based autologous vaccines against relapsed NHL. Although the clinical response was observed only in one third of patients, it correlated with the ability of malignant cells to undergo ICD (61). Similar approach has been already tested in CLL. Partial response, manifested by enhanced T cell proliferation and increase in a co-stimulatory molecule CD80 in CLL cells, was detected in one third of patients and correlated with lack of disease progression (62). Overall, the observed response to oxidized vaccines was relatively weak and the follow-up clinical trials have not been undertaken.

Apart from the direct cytotoxic effects on cancer cells and a positive impact on the immune response by inducing ICD, ROS can affect the course of the disease and therapy outcome by directly interacting with immune cells. Numerous findings accumulated over years (summarized in Table 1) have identified various effects of high ROS levels on different populations of immune cells. The chronic oxidative stress observed in B-cell malignancies may contribute to the immunosuppressive microenvironment and be a component of the immune evasion by malignant B-cells. Furthermore, it may negatively affect the efficacy of immunotherapies involving immune cell populations amenable to oxidative stress-mediated dysfunctions (e.g., therapeutic monoclonal antibodies, cellular therapies with autologous cytotoxic immune cells). The effects of oxidative stress on immune cells were not comprehensively studied in B-cell malignancies so far and the investigations are mostly limited to CLL. NK cells are the population of innate immune cells with remarkable sensitivity to negative ROS effects (73). CLL patients with higher ROS levels had elevated proportion of CD56^bright^CD16^dim^ NK cells in their circulation (4), the subpopulation which is characterized by significantly smaller cytotoxic activity (81). Alterations in conventional T lymphocytes were also observed in CLL patients with high ROS levels. Both CD4^+^ and CD8^+^T cells had reduced expression of CD3ζ, a key subunit of T cell receptor. Additionally, CD4^+^T cells' activation markers (CD69, HLA-DR, and CD137) were decreased. When CD4^+^T cells were *in vitro* stimulated for proliferation and activation in the presence of primary CLL cells, the addition of a ROS scavenger, N-acetylcysteine, significantly increased the expression of the activation markers and IFNy production in the T cells (4).

**Table 1:** Effects of excessive ROS levels on different populations of immune cells.

**Immune cell subset**	**ROS effects**
Macrophages	M2 polarization (63–65) Induction of immunosuppressive phenotype (66) Release of immunosuppressive chemokines (66)
MDSC	Maintaining undifferentiated, immunosuppressive phenotype (67–69)
Dendritic cells	Impaired antigen presentation by DCs (70)
NK Cells	Impaired activation and degranulation (71) Decreased cytotoxicity (72) Induction of apoptosis (73, 74)
Cytotoxic T-Cells	Promoting mitochondrial exhaustion of CD8^+^T-Cells (75) Suppression of T-cell responses (76) Induction of apoptosis (77)
Regulatory T-Cells	Treg accumulation in the tumor microenvironment (78) Inducing adenosine-mediated immunosuppression (79) Better survival under oxidative stress (80)

The oxidative imbalance also entails changes in other T cell subpopulations. In CLL patients it is associated with reduced number of memory T cells and a shift toward Th9 cells. The Th9 cells are a subpopulation of CD4^+^T cells, characterized by the secretion of IL-9, the cytokine linked with leukemogenesis (82). Tregs are another CD4^+^T cell subpopulation which is enriched in B-cell malignancies (83, 84). In CLL, the increase in Tregs correlated with disease progression (85). It was shown that Tregs survive better in the highly oxidative TME than conventional CD4^+^ T cells (80), yet it remains to be elucidated whether the accumulation of Tregs in B-cell malignancies is related to increased oxidative stress.

Another important question is how treatment-related oxidative stress, which is stronger but more transient, affects antitumor immune response. It was demonstrated that anti-CD20 therapeutic monoclonal antibodies, rituximab and ofatumumab, induced potent ROS release from human monocytes cultured *in vitro* in the presence of primary CLL cells (72). Importantly, the mAbs triggered NK cell apoptosis when the NK cells were co-cultured with ROS-producing monocytes, reducing the efficacy of antibody-dependent cellular cytotoxicity (ADCC) and antitumor efficacy (72). These results suggest that the immunosuppressive ROS may also be generated in CLL patients during mAbs immunotherapy and may diminish its efficacy. Indeed, accumulation of immunosuppressive myeloid cell populations were detected both in murine model of CLL as well as in CLL patients (30).

## Concluding Remarks

In recent years, a growing number of studies reports dysregulated redox homeostasis in various B-cell malignancies and the unique vulnerability of malignant B-cells to undergo oxidative stress-mediated apoptosis. We observe significant progress in our understanding of key antioxidant pathways in specific subtypes of B-cell malignancies. As exaggerated oxidative stress leads to apoptosis, this may be therapeutically utilized, as it renders malignant B cells susceptible to antioxidant system inhibitors and other prooxidant treatments.

Although prooxidant therapeutic approaches have already proved effective in preclinical models of B-cell malignancies, their effective use in patients requires further research. This is mainly caused by the multifaceted effects of ROS on the immune system. On one hand, ROS trigger ICD of malignant B cells, which has been utilized in the production of autologous vaccines against CLL and lymphomas. ICD may also contribute to the overall effectiveness of prooxidant therapies used *in vivo*, due to the stimulation of the antitumor immune response. However, prolonged exposure to high ROS levels may negatively affect the viability and function of immune effector cells, which diminishes antitumor immune response and undermines the effects of therapy. To date, in B-cell malignancies only a few studies confirm that ROS may impair viability and effector functions of NK and T cell. However, it is still poorly understood how oxidative stress contributes to the immunosuppression observed in advanced B-cell malignancies, and even more so, how oxidative stress influences the development of an antitumor immune response as a result of ROS-inducing therapies. Further studies are needed to investigate the effects of ROS elicited by prooxidant therapies on antitumor immune response in B-cell malignancies. These studies should include both immunocompetent and immunodeficient animal models, as well as monitoring the function of immune cells during clinical studies testing the effects of prooxidant therapies in patients with B-cell malignancies.

## Author Contributions

MF developed the idea for the topic of the article. MF, AG, and KD contributed to the final conception, literature search, writing, editing, and critical revision of the manuscript. KD prepared a figure and a table. All authors contributed to the article and approved the submitted version.

## Conflict of Interest

The authors declare that the research was conducted in the absence of any commercial or financial relationships that could be construed as a potential conflict of interest.
